# Female Urethral Diverticulum Carcinoma: A Case Report and Literature Review

**DOI:** 10.1155/2017/8918492

**Published:** 2017-05-25

**Authors:** Ryo Kasahara, Tadashi Tabei, Yukio Tsuura, Kazuki Kobayashi

**Affiliations:** ^1^Department of Urology, Yokosuka Kyosai Hospital, 1-16 Yonegahamadori, Yokosuka, Kanagawa, Japan; ^2^Department of Pathology, Yokosuka Kyosai Hospital, 1-16 Yonegahamadori, Yokosuka, Kanagawa, Japan

## Abstract

A 48-year-old woman with a history of voiding difficulty visited our hospital. Magnetic resonance imaging revealed a periurethral tumor, which was pathologically diagnosed as an adenocarcinoma via transperineal needle biopsy. Radical cystectomy and urethrectomy were performed, and the urinary tract was reconstructed using an ileal conduit. Pathological examination of a resected specimen confirmed adenocarcinoma of the urethral diverticulum. The patient received adjuvant gemcitabine and cisplatin chemotherapy. She is alive at 5 months since the operation.

## 1. Introduction

Urethral malignancies are rare in the female population. We encountered a case of urethral diverticulum cancer in a woman, which was difficult to diagnose and was treated with surgery and chemotherapy. Herein, we describe our experience with this case and review the relevant English literature.

## 2. Case Presentation

**Figure 1:**
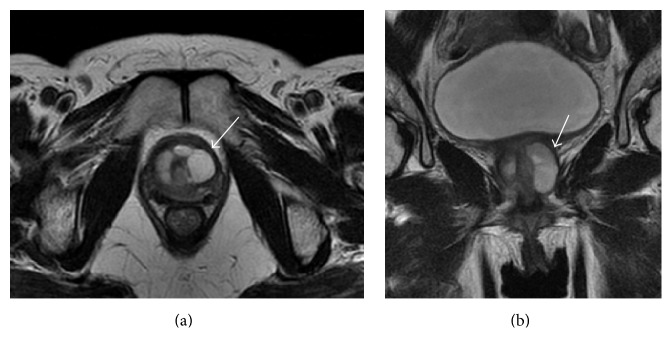
T2-weighted magnetic resonance image of the pelvis. (a) Axial and (b) coronal views of the periurethral diverticulum (arrow).

A 48-year-old woman with a history of voiding difficulty visited a nearby hospital, where she was prescribed urapidil. However, her symptom worsened, and, therefore, clean intermittent catheterization was initiated. For further examination, magnetic resonance imaging (MRI) was performed, which revealed a periurethral diverticulum (Figure 1). The diverticulum was punctured, and a drainage catheter was inserted for 3 days.

**Figure 2:**
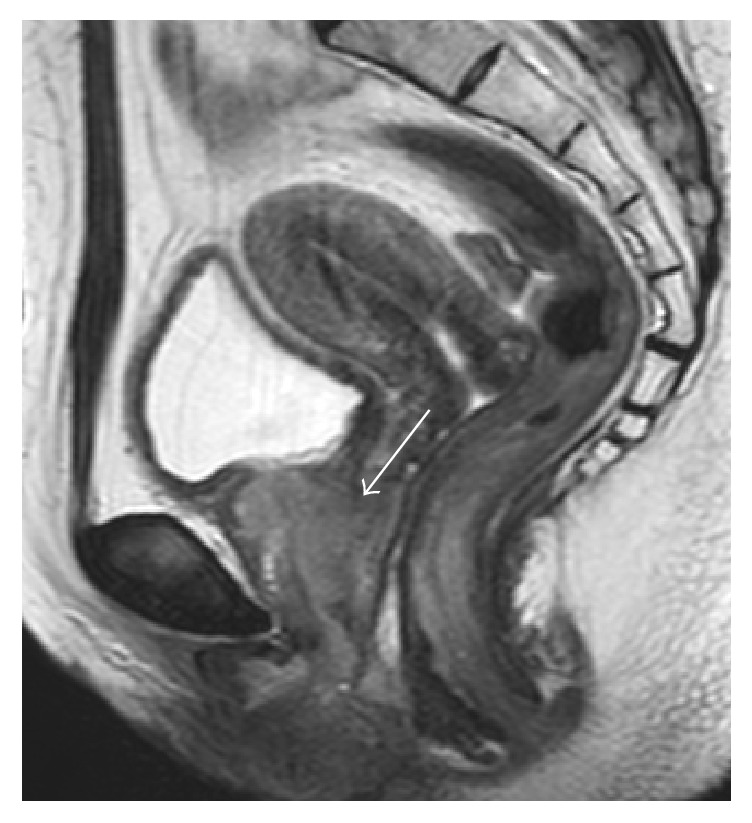
T2-weighted magnetic resonance image of the pelvis. The coronal image shows vaginal invasion of the tumor (arrow).

Three months later, transvaginal ultrasonography showed that the size of the diverticulum had not reduced. Subsequently, she was transferred to our department for further examination and treatment. In order to assess the diverticulum, MRI was performed, which revealed a periurethral tumor (Figure 2). The tumor had low signal intensity on a T1-weighted image and high signal intensity on a T2-weighted image. It was suspected to be malignant and appeared to invade the vagina. Laboratory data showed elevated carcinoembryonic antigen levels (22.8 ng/mL; normal range, 0–5 ng/mL).

The patient underwent transperineal needle biopsy of the tumor using transvaginal ultrasonography. The pathologic diagnosis was adenocarcinoma, and immunohistochemistry revealed CK7 positivity and CK20 negativity. The tumor cells did not express the estrogen receptor or progesterone receptor. Owing to lack of CDX2 expression, the gastrointestinal region was excluded as a possible site of origin of the tumor. The tumor had smooth muscle tissue and prostate-like tissue and was therefore diagnosed as a urethral cancer.

**Figure 3:**
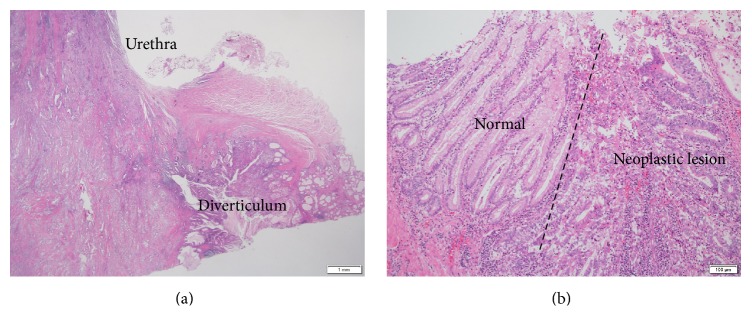
Microscopic findings of the resected specimen. (a) Squamous metaplasia in the urethra and periurethral diverticulum. (b) Normal glandular epithelium of the diverticulum and the neoplastic lesion.

We conducted radical cystectomy and urethrectomy with combined resection of the uterus and vagina. The patient's urinary tract was reconstructed using an ileal conduit. She was discharged from our hospital after 3 weeks without any severe complications. Pathological examination of a resected specimen revealed adenocarcinoma of the urethral diverticulum (Figure 3). The urethra demonstrated squamous metaplasia owing to the urethral catheter or clean intermittent catheterization. The adenocarcinoma was suspected to have originated from the diverticulum because the diverticulum was composed of glandular epithelium. Lymph vessel invasion and vascular invasion were also detected. The tumor invaded the vagina until just under the epithelium, and the infiltration pattern was determined as INFc. The resected specimen demonstrated bilateral external iliac lymph node metastasis and left common iliac lymph node metastasis. The pathologic diagnosis was urethral diverticulum adenocarcinoma pT4pN2 (stage IV).

The patient received 2 courses of adjuvant chemotherapy, comprising gemcitabine and cisplatin, as multidisciplinary therapy. Five months after surgery, she was free of recurrence and metastasis.

## 3. Discussion

Urethral malignancies are rarely reported in the female population, accounting for only 0.02% of all malignancies [1]. According to the National Cancer Institute Surveillance, Epidemiology, and End Results database, adenocarcinomas account for 33% of female urethral diverticulum cancers, with the 2 other pathologic types, squamous cell carcinoma, and transitional (urothelial) cell carcinoma, accounting for 35% and 33%, respectively [2]. However, a previous study reported that most tumors occurring in the diverticulum of the female urethra are adenocarcinomas, such as in the present case [3].

 Grigsby examined the records of 44 women with carcinoma of the urethra (disease stages T1 [*n* = 8], T2 [*n* = 5], T3 [*n* = 22], and T4 [*n* = 9]) [4]. They found that treatment involved surgery in 7 cases, radiotherapy in 25, and a combination of surgery and radiotherapy in 12. However, tumors recurred in 27 women, and the 5-year overall survival was 42%. Their study clarified that tumor size and histology were independent prognostic factors for survival and local tumor control. Particularly, none of the 13 patients with adenocarcinoma were alive 5 years after treatment.

Ahmed et al. surveyed the data for 75 cases of urethral diverticulum cancer (74 involving women and 1 involving a man) [5]. Among the 17 cases of stage T3 cancer, 3 involved local recurrence and 6 resulted in death due to the cancer. Moreover, both cases of stage T4 cancer resulted in death due to the cancer 1 year after surgery. This study claims that locally advanced urethral diverticulum cancer has a poor prognosis, and patients with this disease require multidisciplinary therapy accompanied by extensive resection.

Based on the factors in Table 1, adenocarcinoma of the female urethral diverticulum is thought to have a relatively poor prognosis. The European Association of Urology guidelines for primary urethra carcinoma recommend cisplatin-based chemotherapy [6]. In the present case, the patient's postoperative pathological diagnosis revealed lymph node metastases; therefore, we administered adjuvant gemcitabine and cisplatin chemotherapy. Nonetheless, if lymph node metastasis is clearly detected via preoperative computed tomography (CT) or MRI, neoadjuvant or perioperative chemotherapy is recommended [7].

The patient presented with the chief complaint of voiding difficulty. In general, voiding difficulty among the female population is typically associated with a neurogenic disorder such as spinal cord injury, a neurogenic bladder due to diabetes mellitus, or impaired activity of daily living. Some cases are caused by a gynecological tumor or pelvic organ prolapse. Urethral tumors are rare and are thus likely to be overlooked. Therefore, we recommend imaging analysis via MRI or CT for further examination of voiding difficulty in relatively young female patients.

## Figures and Tables

**Table 1:** Literature summary.

Reference	Number of patients	Results
SEER database (Swartz et al.) [2]	1615	Maximum incidence rate: 9.5 per million women UC: 55%, SCC: 21.5%, adenocarcinoma: 16.4%

Clayton et al. [3]	59	UC: 29%, SCC 15%, adenocarcinoma 56% T1: 18%, T2-3: 69%, T4: 13% (of the 39 patients with known stages) Local resection: 37%, radiation or cystectomy: 63% Tumor recurrence: 41%

Grigsby [4]	44	T1: 18%, T2: 11%, T3: 50%, T4: 20% Surgery: 16%, radiation: 57%, surgery and radiation: 27% 5-year overall survival: 42%

Ahmed et al. [5]	75	T1: 16.7%, T2: 28.6%, T3: 47.6%, T4: 4.8% (of the 42 patients with known stages) Local resection: 38%, radiation: 21%, cystectomy: 40% Local recurrence: 3, death: 6 (of the 17 T3 cases)

SEER, National Cancer Institute Surveillance, Epidemiology, and End Results; UC, urethral cancer; SCC, squamous cell carcinoma.
